# Use of Ambient AI Scribes to Reduce Administrative Burden and Professional Burnout

**DOI:** 10.1001/jamanetworkopen.2025.34976

**Published:** 2025-10-02

**Authors:** Kristine D. Olson, Daniella Meeker, Matt Troup, Timothy D. Barker, Vinh H. Nguyen, Jennifer B. Manders, Cheryl D. Stults, Veena G. Jones, Sachin D. Shah, Tina Shah, Lee H. Schwamm

**Affiliations:** 1 Department of Medicine, Yale School of Medicine, New Haven, Connecticut; 2 Office of the Chief Wellness Officer, Yale New Haven Health–Yale Medicine, New Haven, Connecticut; 3 Department of Biomedical Informatics and Data Science, Yale School of Medicine, New Haven, Connecticut; 4 Abridge AI, Inc, Pittsburg, Pennsylvania; 5 Clinical Excellence Division, CHRISTUS Health, Irving, Texas; 6 Office of the System Medical Director Chief Medical Information Officer of Ambulatory Care, CHRISTUS Health, Irving, Texas; 7 Department of Family Medicine, MemorialCare Health System, Huntington Beach, California; 8 Department of Clinic Informatics, MemorialCare Health System, Huntington Beach, California; 9 Department of Surgery, The Christ Hospital Health Network, Cincinnati, Ohio; 10 Center for Health System Research, Sutter Health, San Francisco, California; 11 Department of Digital Health, Sutter Health, San Francisco, California; 12 Office of the Chief Medical Information Officer, Sutter Health, San Francisco, California; 13 Departments of Medicine and Pediatrics, University of Chicago, Chicago, Illinois; 14 Office of the Chief Medical Information Officer, Biological Sciences Division, University of Chicago Medicine, Chicago, Illinois; 15 Division of Pulmonary and Critical Care, RWJBarnabus Health, Newark Beth Israel Medical Center, Newark, New Jersey; 16 Department of Bioinformatics and Data Sciences, Yale School of Medicine and Digital and Technology Solutions, Yale New Haven Health System, New Haven, Connecticut

## Abstract

**Importance:**

While in short supply and high demand, ambulatory care clinicians spend more time on administrative tasks and documentation in the electronic health record than on direct patient care, which has been associated with burnout, intention to leave, and reduced quality of care.

**Objective:**

To examine whether ambient AI scribes are associated with reducing clinician administrative burden and burnout.

**Design, Setting, and Participants:**

This quality improvement study used preintervention and 30-day postintervention surveys to evaluate the use of the same ambient AI platform for clinical note documentation among ambulatory care physicians and advanced practice practitioners of 6 academic and community-based health care systems across the US. Clinicians were recruited by the health systems’ digital health leaders; participation was voluntary. The study was conducted between February 1 and October 31, 2024.

**Exposure:**

Use of an ambient AI scribe for 30 days.

**Main Outcomes and Measures:**

The primary outcome was change in self-reported burnout, estimated using hierarchical logistic regression. Secondary outcomes of burnout evaluated were changes in note-related cognitive task load, focused attention on patients, patient understandability of notes, ability to add patients to the clinic schedule if urgently needed, and time spent documenting after hours. Outcome measures were linearly transformed to 10-point scales to ease interpretation and comparison. Differences between preintervention and postintervention scores were determined using paired *t* tests.

**Results:**

Of the 451 clinicians enrolled, 272 completed the preintervention and postintervention surveys (60.3% completion rate), and 263 with direct patient care in ambulatory clinics (mean [SD] years in practice, 15.1 [9.3]; 141 female [53.6%]) were included in the analysis. The sample included 131 primary care practitioners (49.7%), 232 attending physicians (88.2%), and 168 academic faculty (63.9%). After 30 days with the ambient AI scribe, the proportion of participants experiencing burnout decreased significantly from 51.9% to 38.8% (odds ratio, 0.26; 95% CI, 0.13-0.54). On 10-point scales, the ambient AI scribe was associated with significant improvements in secondary outcomes of burnout (mean [SE] difference, 0.47 [0.12] points), note-related cognitive task load (mean [SE] difference, 2.64 [0.13] points), ability to provide undivided attention (mean [SE] difference, 2.05 [0.18] points), patient understandability of their care plans from reading the notes (mean [SE] difference, −0.44 [0.17] points), ability to add patients to the clinic schedule if urgently needed (mean [SE] difference, 0.51 [0.24] points), and time spent documenting after hours (mean [SE] difference, 0.90 [0.19] hours).

**Conclusions and Relevance:**

This multicenter quality improvement study found that use of an ambient AI scribe platform was associated with a significant reduction in burnout, cognitive task load, and time spent documenting, as well as the perception that it could improve patient access to care and increase attention on patient concerns in an ambulatory environment. These findings suggest that AI may help reduce administrative burdens for clinicians and allow more time for meaningful work and professional well-being.

## Key Points

**Question:**

What is the association of using ambient artificial intelligence (AI) scribes with clinician administrative burden, burnout, time documenting after hours, and time and attention for patients?

**Findings:**

This quality improvement study of 263 physicians and advance practice practitioners across 6 health care systems found that after 30 days with an ambient AI scribe, burnout among those working in ambulatory clinics decreased significantly from 51.9% to 38.8%. There were also significant improvements in the cognitive task load, time spent documenting after hours, focused attention on patients, and urgent access to care.

**Meaning:**

These findings suggest that AI may have promising applications to reduce administrative burdens for clinicians and allow more time for meaningful work and professional well-being.

## Teaser

This quality improvement study examines whether ambient artificial intelligence (AI) scribes are associated with reductions in clinician administrative burden and burnout.

## Introduction

Large language models are generative artificial intelligence (AI) systems that can produce professional appearing text. They are taught to listen, instantaneously transcribe, assimilate, and assemble a document, with fine-tuning by human training.^1^ Ambient AI platforms can listen to a clinical encounter and draft clinical documentation. This technology has the potential to reduce professional burnout associated with excessive time spent documenting in the electronic health record (EHR) and free professionals for more meaningful time with patients, with loved ones, or for self-care.^2^

Physicians, who are in short supply and high demand,^3^ spend more than half their workday documenting in the EHR,^4,5,6,7^ and only a quarter of their time is spent face to face with patients.^6^ The proportion of time spent documenting continues to escalate,^5,8^ especially for primary care professionals,^9,10,11^ and is associated with burnout, reduction in work effort, and turnover.^10,12,13,14^

The National Academy of Medicine convened a meeting in December 2024 on the potential for AI to improve health worker well-being (eg, reduce burnout).^15^ To date, there are scant, mostly single-center data assessing whether this technology could reduce administrative burden, liberate time for patients, and reduce professional burnout.^16^

The aim of this preintervention and postintervention study was to examine whether 30 days of using an ambient AI scribe is associated with a reduction in burnout among clinicians delivering care in ambulatory clinics. The secondary aims were to explore whether the ambient AI scribe was associated with improvements in cognitive task load, time spent documenting after hours, undivided attention on patients, notes that patients can understand, and adding patients to the clinic schedule if urgently needed.

## Methods

### Participants, Setting, and Intervention

This quality improvement study was conducted between February 1 and October 31, 2024, in 6 health systems across the US that deployed the Abridge ambient AI scribe (Abridge AI, Inc) intervention to draft clinical documentation. The Yale University Institutional Review Board determined the study to not be human participant research because it was a secondary analysis of deidentified aggregated survey data originally collected for quality improvement, for which informed consent was not required. The authors who conducted the statistical analysis (K.D.O. and D.M.) were not involved in the intervention; had no contact with participants; and received no incentives or remuneration from the vendor. The study followed the Standards for Quality Improvement Reporting Excellence (SQUIRE) reporting guideline.

Participants agreed to complete an evaluation before and after the 30 days of ambient AI scribe use. Health systems’ digital health leaders recruited ambulatory care medical doctors and advanced practice practitioners to participate. Participation was voluntary without incentives other than the potential benefit of the ambient AI scribe. Participants were onboarded by their organization with standard materials from the vendor and site-developed training methods at their discretion. Participants received a preintervention survey and a postintervention survey 30 days later (eTable 1 in Supplement 1).

For use of the ambient AI scribe, clinicians selected the relevant patient encounter from their ambulatory EHR schedule, obtained verbal consent from the patient, and recorded the encounter. After recording, documentation was instantaneously generated in a standard medical office note format on a secure online portal that allowed viewing and editing. Clinicians could highlight segments of the note to see underlying transcripts or hear source audio recordings. After editing, the text was automatically imported into the clinician’s note template. Patients were informed that after a short grace period, the original recordings and associated transcripts would be erased. The vendor confirmed that all sites used the same version of the technology throughout the 9-month study period.

The vendor distributed the standardized survey before the intervention and after day 30 of the intervention for 5 organizations; the sixth system distributed it independently. Participation was not anonymous; individuals were prompted by the site-based team to complete assessments. Participants were included in the analysis if they practiced in an ambulatory clinic and completed the preintervention and postintervention surveys. Aggregated and deidentified data from all 6 sites were sent to independent investigators at 1 of the participating sites (K.D.O. and D.M.) for analysis (Figure; eFigure 1 in Supplement 1).

**Figure.**
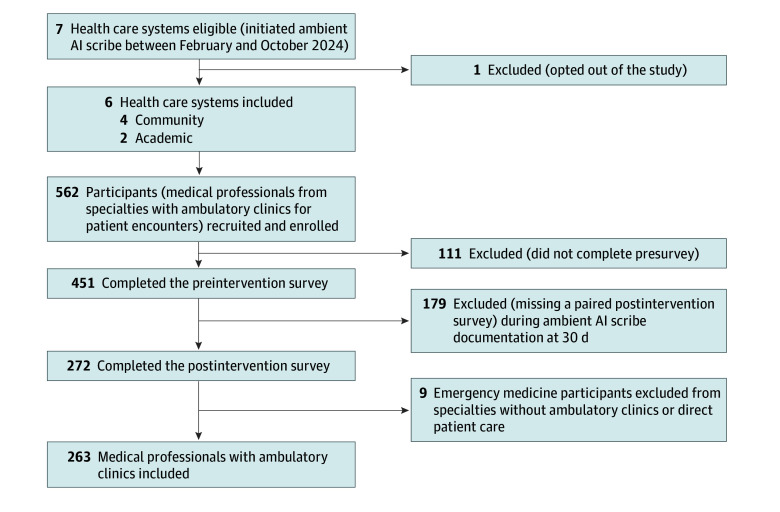
Inclusion and Exclusion Criteria AI indicates artificial intelligence.

### Measures

#### Primary Outcome

Burnout was assessed with a 5-point, single-item metric that has been validated against the emotional exhaustion domain of the full Maslach Burnout Scale.^17,18,19^ The single-item metric is part of the popular Mini-Z scale,^19^ often used for brief surveys. Per standard convention, burnout was defined by a score of at least 3 points, which allowed for comparison with the existing literature in which 3 is assigned to, “I am beginning to burn out and have 1 or more symptoms of burnout (eg, emotional exhaustion).”^17,18,19^

#### Secondary, Exploratory Outcomes

We evaluated several factors important to clinicians for an association with the use of ambient AI scribes. Note-related cognitive task load was assessed by a sum composite score of 3 pertinent items modified from the validated 6-item National Aeronautical and Space Administration Task Load Index.^20,21,22^ A 4-item version of this scale (excluding constructs similar to burnout, including frustration and performance) has been used previously to assess a national sample of physicians.^21^ This note-related, 3-item version excludes evaluation of physical demand and includes the questions, “How mentally demanding is it to write your notes,” “how hurried/rushed is the pace of your note writing,” and “how hard do you have to work to accomplish your level of note-writing performance?” Focused attention was assessed by the statement, “I’m able to give patients my undivided attention during the encounter,” on a scale of 1 (strongly disagree) to 5 (strongly agree). Patient access was assessed by the statement, “I feel that I could add at least 1 more patient encounter to my clinic session if urgently needed,” on a scale of 1 (strongly disagree) to 5 (strongly agree). Number of patients to be urgently added to the clinic schedule was assessed by the statement, “I estimate the number of patient encounters I could add to my clinic session is 1 patient, 2 patients, 3 patients, or 4 or more patients.” Documentation after hours was assessed by the statement, “The average amount of time I spend per week writing notes outside of clinic hours is,” selected from a range of 1 to 10 hours (1 site allowed free numerical entry, which was truncated after 10 hours for analysis). For post intervention, the same questions and statements were surveyed, prefaced by the words, “With Abridge,” to assess the influence of the ambient AI scribe in the note-writing task (eTable 1 in Supplement 1).

### Statistical Analysis

The preintervention and postintervention analysis used aggregated, deidentified survey data collected as part of a quality improvement program evaluation across 6 health care systems deploying the same version of an ambient AI scribe. The statistical analysis plan was preregistered, including a commitment to report null findings.^23^ The primary outcome was burnout, and secondary analyses included changes in multiple other outcome measures linearly transformed to 10-point scales for ease of interpretation and comparison. The sample was characterized by standard descriptive statistics (including sex, years in practice, and specialty). Race and ethnicity data were not collected to mitigate the risk of identifying respondents even after otherwise aggregating and deidentifying data for secondary analysis.

For the primary outcome of burnout, we used the conventional dichotomized burnout outcome (score ≥3). For the primary analysis, we regressed clinicians’ burnout indicators on the intervention period indicator (preintervention vs postintervention) using hierarchical logistic regression that included random intercepts for clinicians nested in sites (ie, 1 observation per clinician per time point). A post hoc sensitivity analysis with a burnout cutoff of at least 4 rather than at least 3 was also conducted to assess changes in severe burnout. Paired *t* tests on unadjusted 10-point scales were used for exploratory investigation of secondary outcomes and subgroup effects across clinician demographic traits, including practice model, degree, specialty, years in practice, and sex. Consistent with our directional hypothesis, statistical significance was set at *P* < .05 (1-sided). There were no corrections for multiple comparisons or tests of collinearity as these were purely exploratory in nature. Analyses were conducted on complete datasets; missing data were not imputed. Site 5 (which included 63 participants) did not include survey burnout questions and so was censored from the primary outcome (eTable 2 in Supplement 1 shows a comparison of clinician demographics with the other sites). Site 4 (which included 19 participants) did not include patient access questions, and at 1 site, the number of hours outside of work was converted from free text to the ordinal scale to harmonize the data. All analyses were performed using Stata/MP, version 18.5 (StataCorp LLC).

## Results

Of 451 participants, 272 completed both surveys (60.3% completion rate), and after excluding 9 emergency medicine participants without specialized ambulatory clinics or direct patient care, 263 clinicians were included in the study (mean [SD] years in practice, 15.1 [9.3]; 141 female [53.6%], 120 male [45.6%], and 2 unreported sex [0.8%]) (Figure; Table 1). These individuals included 131 primary care professionals representing general internal medicine, family practice, internal medicine/pediatrics, and pediatrics (49.7%), 46 adult specialists (17.5%), 14 working in neurology and psychiatry (5.3%), and 72 working in surgical specialties (27.4%). The sample included predominantly attending physicians (232 [88.2%]) and academic faculty (168 [63.9%]). Minus academic site 5, the sample of 194 who provided burnout data was similar to the larger sample, with most being attending physicians (179 [92.3%]), academic faculty (99 [51.0%]), and women (108 [55.7%]). The burnout sample had been in practice for fewer years (mean [SD], 13.0 [8.2] years) and had fewer adult specialists (28 [14.4%]). Eight of the same respondents (4.2%) were missing data on practice model, specialty, and sex (eTable 2 in Supplement 1).

**Table 1.** Demographics of 263 Participants Completing the Preintervention and Postintervention Surveys

Characteristic	Participants, No. (%)
Health system site	
1	44 (16.7)
2	17 (6.5)
3	9 (3.4)
4	19 (7.2)
5	69 (26.2)
6	105 (39.9)
Clinician type	
Medical doctor	232 (88.2)
Advanced practice practitioner	29 (11.0)
Unknown	2 (0.8)
Practice model	
Academic	168 (63.9)
Medical group employed	90 (34.2)
Community private practice	5 (1.9)
Specialty	
Family practice or internal medicine/pediatrics	55 (20.9)
Adult general internal medicine	38 (14.4)
Adult specialty care	46 (17.5)
Pediatrics	38 (14.4)
Neurology or psychiatry	14 (5.3)
Obstetrics and gynecology	27 (10.3)
Surgery	45 (17.1)
Experience level	
Years in practice, mean (SD)	15.1 (9.3)
Years in practice by group	
≥1 to ≤5	44 (16.9)
>5 to ≤10	46 (17.6)
>10 to ≤15	69 (26.4)
>15 to ≤20	36 (13.8)
>20	66 (25.3)
Sex	
Female	141 (53.6)
Male	120 (45.6)
Not reported	2 (0.8)

Among all participants, 252 (95.9%) generated at least 5 notes using the ambient AI scribe. Prior to the intervention, participants performed clinical documentation using manual typing (218 [82.9%]), templates or dot phrases (224 [85.2%]), dictation (123 [46.8%]), or human scribes (43 [16.3%]). Only 4 participants (1.5%) had previous experience with another ambient AI scribe solution.

Among 186 participants included in the burnout models, the proportion with the primary outcome of burnout (using the standard cutoff of ≥3) decreased from 51.9% to 38.8% (difference, 13.1 percentage points; SE, 3.3 percentage points; 95% CI, 6.5-19.7 percentage points), corresponding to an adjusted odds ratio of burnout of 0.26 (95% CI, 0.13-0.54; *P* < .001) after adjustment for clinician demographic covariates and clinicians nested in sites (Table 2). A post hoc sensitivity analysis using a severe burnout cutoff of at least 4 showed an adjusted reduction in the proportion with severe burnout from 18.4% to 12.2% (difference, 6.2 percentage points; SE, 2.5 percentage points; 95% CI, 1.3-11.2 percentage points; *P* = .01).

**Table 2.** Univariable and Multivariable Models of the Association of the Intervention With Self-Reported Burnout

Model^a^	No. of participants	Mean (SE), %		Difference, percentage points	OR (95% CI)	*P* value
		Baseline	Follow-up			
Univariable	186	51.4 (4.1)	37.5 (4.1)	−13.9 (3.7)	0.30 (0.15-0.59)	<.001
Multivariable^b^	184	51.9 (3.3)	38.8 (3.3)	−13.1 (3.3)	0.26 (0.13-0.54)	<.001

Abbreviation: OR, odds ratio.

^a^ Hierarchical mixed-effects logistic regression models with random intercepts for clinicians nested in sites.

^b^ Multivariable models adjusted for degree, practice model, specialty, years in practice, sex, and site.

After 30 days of ambient AI scribe use, participants also experienced significant improvement in all but 1 of the secondary exploratory factors assessed by unadjusted paired *t* tests with outcomes normalized to continuous 10-point scales: note-related cognitive task load (mean [SE] difference, 2.64 [0.13] points; *P* < .001), ability to focus undivided attention on patients (mean [SE] difference, −2.05 [0.18] points; *P* < .001), ability to add patients to the clinic schedule if urgently needed (mean [SE] difference, −0.51 [0.24] points; *P* = .02), create notes that patients can understand (mean [SE] difference, −0.44 [0.17] points; *P* = .005), and reduce time spent documenting after hours (mean [SE] difference, 0.90 [0.19] hours; *P* < .001) (Table 3).

**Table 3.** Comparison of Secondary Outcome Measures Before and After Use of the Ambient AI Scribe

Outcome	No. of participants	Mean (SE) score^a^			*P* value
		Baseline	Follow-up	Difference	
Burnout	186	4.59 (0.15)	4.12 (0.15)	0.47 (0.12)	<.001
Note-related cognitive task load					
Any	243	7.10 (0.09)	4.46 (0.12)	2.64 (0.13)	<.001
Temporal demand	249	7.01 (0.11)	4.35 (0.13)	2.66 (0.16)	<.001
Effort	248	7.31 (0.12)	4.71 (0.13)	2.60 (0.15)	<.001
Mental demand	254	6.84 (0.12)	4.38 (0.15)	2.46 (0.15)	<.001
Documentation after hours	263	4.95 (0.18)	4.05 (0.16)	0.90 (0.19)	<.001
Focused attention on patients	253	6.51 (0.16)	8.56 (0.11)	−2.05 (0.18)	<.001
Comprehensible care plans	254	7.34 (0.13)	7.79 (0.13)	−0.44 (0.17)	.005
Agreeable to add urgent patients	230	6.21 (0.21)	6.72 (0.20)	−0.51 (0.24)	.02
No. of additional patients (1 to ≥4)	91	2.19 (0.11)	2.16 (0.11)	0.02 (0.11)	.58

Abbreviation: AI, artificial intelligence.

^a^ Unadjusted preintervention and postintervention paired *t* tests transformed to 10-point scales.

On further exploration using the 10-point scales with unadjusted paired *t* tests, the burnout score across all participants was significantly reduced before vs after intervention from 4.59 to 4.12 points (mean [SE] difference, 0.47 [0.12] points; *P* < .001). Several subgroups had statistically significant reductions in burnout, including medical doctors (mean [SE] difference, 0.52 [0.12]; *P* < .001), participants in academia (mean [SE] difference, 0.32 [0.14] points; *P* = .01), medical group–employed clinicians (mean [SE] difference, 0.65 [0.20] points; *P* = .001), participants in practice for 10 to 15 years (mean [SE] difference, 0.38 [0.22]; *P* = .048), men (mean [SE] difference, 0.48 [0.16] points; *P* = .002), and women (mean [SE] difference, 0.46 [0.17] points; *P* = .004). Among ambulatory specialties, reductions were seen for family medicine and pediatrics (mean [SE] difference, 0.98 [0.28] points; *P* < .001), obstetrics and gynecology (mean [SE] difference, 0.59 [0.34]; *P* = .048), and adult specialties (mean [SE] difference, 0.50 [0.28] points; *P* = .04) (eTable 3 in Supplement 1).

## Discussion

This quality improvement study is, to our knowledge, the first large, multicenter preintervention and postintervention evaluation to assess the association of ambient AI scribes with clinician experience. After 30 days with the ambient AI scribe, 74% lower odds of participants experiencing burnout was found. Controlling for organizational and demographic factors, the proportion of participants reporting burnout decreased from 51.9% to 38.8%. Compared with baseline, implementation of the ambient AI scribe was associated with increased attention on patients, clinician confidence that patients understood care plans from reading the notes, and agreement that additional patients could be added to the clinic schedule if urgently needed, all while reducing note-related cognitive task load and the time spent documenting after hours.

While the high prevalence of documentation burden and its associations with burnout are well known,^10,24,25,26,27^ there have been few intervention studies reported.^28,29^ The existing small, single-center, preintervention and postintervention evaluations of in-person or remote human scribes and ambient AI scribes reported that scribing reduced the documentation burden for physicians, improved note comprehension for patients, facilitated focused attention on patients, and improved professional well-being^30,31,32,33,34,35,36^ but did not reliably decrease the time spent documenting after hours or increase the ability to add more patients to the schedule.^27,37^

The decreases in burnout we observed are comparable to what has been reported in studies of human scribes and ambient AI scribes. Our study found 74% lower odds of burnout after 30 days with the first iteration of this ambient AI platform. Two smaller, single-center studies evaluating an ambient AI scribe at 5 weeks and 3 months found similar reductions in burnout using a different scale that was not directly comparable to the single item used here.^31,33^ A study of 37 physicians in primary care using the same single-item burnout metric found an 85% reduction in the odds of burnout using remote human scribes,^30^ but the period of 2019 to 2020 made the comparison difficult given that physician burnout was dynamic during the COVID-19 pandemic.^38,39,40^

Our study reported a 2.64-point reduction on a 10-point scale in note-related cognitive task load. Using different ambient AI scribes, others found similar statistically significant reductions in cognitive task load of 24.42 points on a 100-point scale.^31^ Our participants reported the equivalent of 10.8 minutes saved per workday after intervention. Prior studies of a different ambient AI tool found that afterhours work declined by 5.17 minutes per day after 3 months,^32^ with no significant reduction in afterhours work after 180 days based on EHR data.^34^ In comparison, a 3-month pilot study using remote human scribes reduced afterhours documentation by 1.1 minutes per scheduled patient encounter (*P* = .004),^35^ which is equivalent to 22 minutes per day for an average clinic day containing 20 encounters. Lack of comparable metrics makes comparisons of these studies difficult to interpret. There are no agreed-upon standards on which to compare note quality, yet the statistically significant increase in confidence that patients would understand their care plans by reading the ambient AI–generated note is consistent with various other smaller studies of scribes.^31,33,36^

Standard metrics and methods are needed to definitively assess and compare quality improvement, especially as AI technologies are introduced in health care.^27,41,42^ The American Medical Association Joy in Medicine Recognition Program recommends measuring and tracking standard EHR metrics by specialty and care setting, normalized to 8 hours of work per day, including total EHR time, time on encounter note documentation, time on inbox, and work outside of work.^42^ Using these metrics, researchers have compared the number of patient-scheduled hours resulting in a 40-hour workweek by specialty; ambulatory specialties (eg, infectious diseases, geriatrics, hematology, primary care) have shown the lowest proportion of the workday available for patient-scheduled hours, largely owing to the excessive time spent documenting and completing EHR tasks.^11^ Standard metrics allowed researchers to track and report on the escalation of professional time spent on EHR administrative tasks that now consume more than half of professionals’ time, and documenting the clinical encounter itself is only a fraction of that time.^4,5,6,8,12,43^ Much of this increase was associated with health care reform, pandemic-initiated telehealth and health portal adoption, open access to notes, and policies requiring computer-physician order entry. These factors may explain why scribe-assisted encounter documentation is associated with only modest time savings, highlighting the need for future support of additional EHR tasks.

Despite these small changes in documentation time, the significant change in burnout suggests that these small improvements may have an outsized influence or that other aspects of the intervention may improve overall clinician experience. Clinicians in ambulatory care have the greatest documentation burden^11^ and stand to gain the most from documentation assistance. Unlike surgery or procedures, ambulatory care is primarily cognitive and requires focused attention on patients to facilitate complex medical decision-making, patient education, and establishing a trusting therapeutic relationship to promote adherence to recommended treatment plans.^44,45,46^ Our findings suggest that AI scribes are associated with a more satisfying, patient-centered experience that is central to professional satisfaction and protective against burnout.^44,45,46,47,48^ The time saved documenting after hours frees time for self-care,^49^ frees time with loved ones,^50^ and contributes to work-life satisfaction.^2,51^ Physician groups with low burnout rates are associated with higher quality care,^52,53^ retention of physicians committed to full-time work,^54,55,56,57^ avoidance of the average cost of turnover of $800 000 to $1.3 million per physician lost,^54,58^ and the excess health care costs attributed to disrupting continuity of care between physician and patients^59^ (eFigure in Supplement 1).

### Limitations

There are limitations to this study. The included health care organizations implemented the ambient AI scribe as a quality improvement initiative; as such, the evaluation was not designed for research purposes, and the dataset is one of convenience. The baseline demographic characteristics of the participating organizations were not available to evaluate whether the sample was representative of respective professional populations or whether self-selection in recruitment and attrition represented a biased perspective. There was no control group to adjust for temporal trends. As this dataset only included complete sets of preintervention and postintervention survey results, we could not characterize noncompleters or nonresponders. It is conceivable that recruitment may have been biased toward individuals in favor of new technologies and more likely to give a favorable review.^60^ The findings were subjective reports of the professional experience and not paired with quantitative data on clinical documentation efficiency from the EHR. These early adopters may have responded favorably to please their digital health leadership, as the survey was not anonymous. We were not able to control for unmeasured confounding. Finally, 1 academic medical center (69 respondents) did not participate in the burnout question; while the burnout sample was grossly similar demographically to the larger sample, the comparative interpretation of the secondary outcomes must be considered exploratory. Overall, the analysis did control for other factors, including diversity in health systems (national sample of academic and community-based sites), professional degrees, specialties, time in practice, and sex. Despite the limitations, the results were favorable in magnitude and statistical significance and consistent with previous smaller studies and may support generalizability to other health system ambulatory clinics.

## Conclusions

This multicenter quality improvement study of 263 ambulatory clinicians found that after 30 days using an ambient AI scribe, the proportion of clinicians with burnout dropped from 51.9% before to 38.8% after the intervention, with associated improvements in the cognitive task load, time spent documenting after hours, focused attention on patients, and urgent access to care. Artificial intelligence scribes may represent a scalable solution to reduce administrative burdens for clinicians and allow more time for meaningful work and professional well-being. Ambient AI solutions may be scalable at a lower cost than human scribes.

## Notes

Supplement 1. **eTable 1.** Survey **eFigure.** Logic Model **eTable 2.** Demographic Statistics: Total Sample Compared With the Burnout Sample (Without Site 5) **eTable 3.** Ten-Point Burnout for Demographic Group, Paired *t* Test Pre and Post Intervention (Means and Difference)

Supplement 2.
Data Sharing Statement
