# Supplementary material for: Use of Ambient AI Scribes to Reduce Administrative Burden and Professional Burnout
Source: JAMA Netw Open. 2025 Oct 2;8(10):e2534976. doi: 10.1001/jamanetworkopen.2025.34976 (PMC12492056; doi:10.1001/jamanetworkopen.2025.34976)
Supplement: Supplement 1. — eTable 1. Survey eFigure. Logic Model eTable 2. Demographic Statistics: Total Sample Compared With the Burnout Sample (Without Site 5) eTable 3. Ten-Point Burnout for Demographic Group, Paired t Test Pre and Post Intervention (Means and Difference) [file jamanetwopen-e2534976-s001.pdf]

Supplemental Online Content

Olson KD, Meeker D, Troup M, et al. Ambient AI scribes to reduce administrative burden and professional burnout. *JAMA Netw Open*. 2025;8(10):e2534976.
doi:10.1001/jamanetworkopen.2025.34976

eTable 1. Survey

eFigure. Logic Model

eTable 2. Demographic Statistics: Total Sample Compared With the Burnout Sample (Without Site 5)

eTable 3. Ten-Point Burnout for Demographic Group, Paired *t* Test Pre and Post Intervention (Means and Difference)

This supplemental material has been provided by the authors to give readers additional information about their work.

© 2025 Olson KD et al. *JAMA Network Open*.

eTable 1. Survey

Content	Question	Response
**Unique Identifier**	Site-Specific unique identifier requested	Free Text
**Baseline Workflow**	My current note-writing workflow is ___	[Select all that apply]<br>- Manual Typing<br>- Templates / dotphrases<br>- Dictation<br>- Other ambient solution<br>- Scribes<br>- Other
**NASA TLX: Mental Demand** (nr-CTL_3m)	On a scale from 0-20, how mentally demanding is it to write your notes?	0-20 selection<br>(very low - very high)
**NASA TLX: Temporal Demand** (nr-CTL_3d)	On a scale from 0-20, how hurried / rushed is the pace of your note writing?	0-20 selection<br>(very low - very high)
**NASA TLX: Effort** (nr-CTL_3e)	On a scale from 0-20, how hard do you have to work to accomplish your level of note-writing performance?	0-20 selection<br>(very low - very high)
**Mini Z: Burnout**	Using your own definition of "burnout", please choose one of the options below:	1 - I enjoy my work. I have no symptoms of burnout<br>2 - I am under stress, and don't always have as much energy as I did, but I don't feel burned out<br>3 - I am beginning to burn out and have one or more symptoms of burnout (e.g. emotional exhaustion)<br>4 - The symptoms of burnout that I'm experiencing won't go away. I think about work frustrations a lot<br>5 - I feel completely burned out. I am at the point where I may need to seek help
**Undivided Attention**	I'm able to give patients my undivided attention during the encounter.	1 - Strongly Disagree<br>2 - Disagree<br>3 - Neutral<br>4 - Agree<br>5 - Strongly Agree
**Work Outside Work**	The average amount of time I spend *per week* writing notes outside of clinic hours is:	time in hours
**Patient Access**	I feel that I could add at least one more patient encounter to my clinic session if urgently needed.	1 - Strongly Disagree<br>2 - Disagree<br>3 - Neutral<br>4 - Agree<br>5 - Strongly Agree

© 2025 Olson KD et al. *JAMA Network Open*.

**Patient Access – Branch**	[ask if *Agree* or *Strongly Agree to Access Question above*]<br>I estimate the number of patient encounters I could add to my clinic session is:	to previous question 1 patient<br>2 patients<br>3 patients<br>>3 patients
**Patient Comprehension**	My notes are as comprehensive and complete as I would like them to be for clinical purposes.	1 - Strongly Disagree 2 - Disagree 3 - Neutral 4 - Agree 5 - Strongly Agree
**Pre-Intervention Feedback**	Do you have any other feedback or thoughts you'd like to share	Free text

© 2025 Olson KD et al. *JAMA Network Open*.

eFigure 1. Logic Model

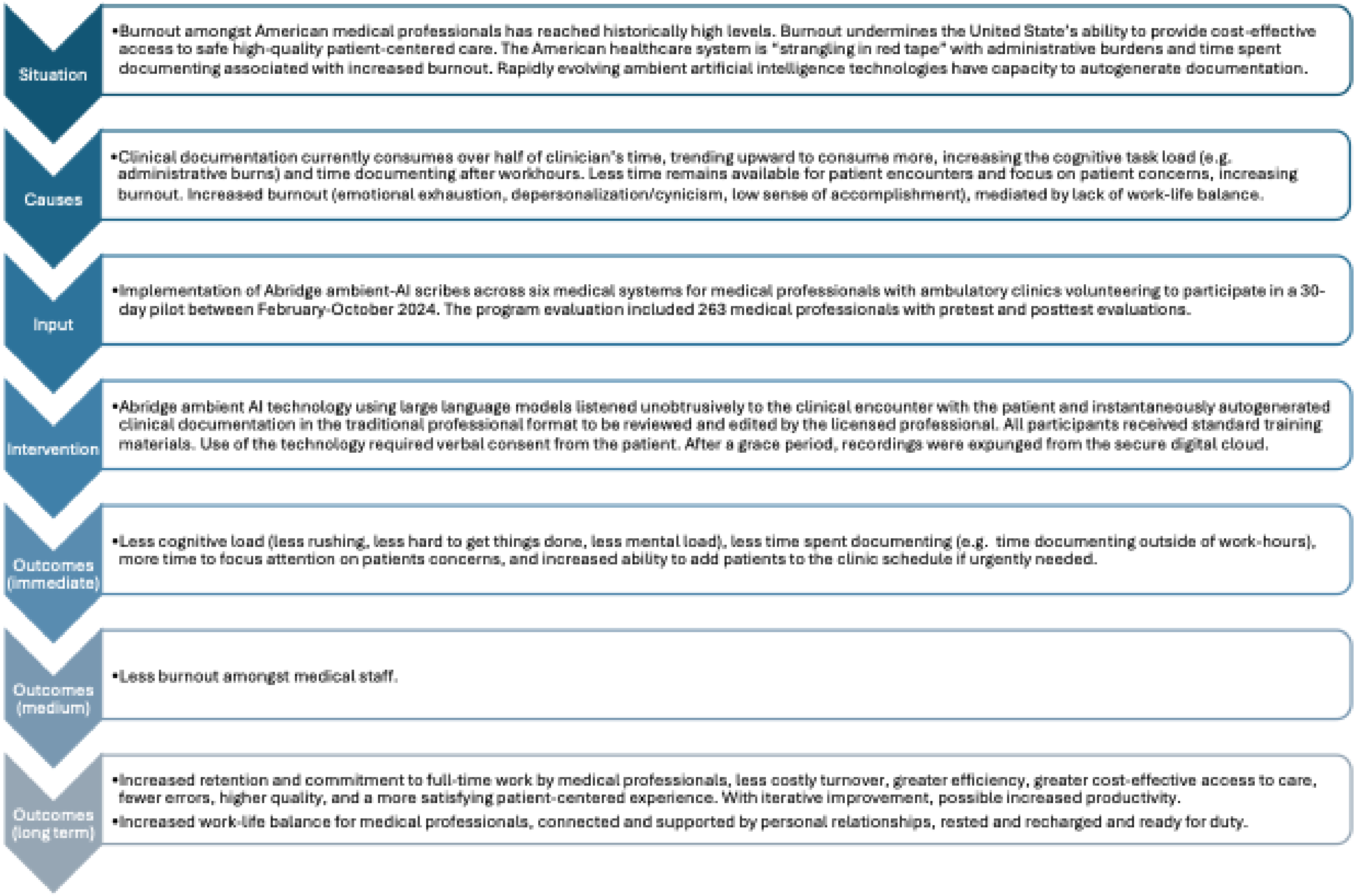

© 2025 Olson KD et al. *JAMA Network Open*.

eTable 2. Demographic Statistics: Total Sample Compared With the Burnout Sample (Without Site 5)

DESCRIPTIVE STATISTICS: DEMOGRAPHICS, BURNOUT ANALYSIS

PRETEST TOTAL SAMPLE			PRETEST BURNOUT-ONLY SAMPLE		
**Health System Site:**			**Health System Site:**		
id	Freq.	Percent	id	Freq.	Percent
1	44	16.73	1	44	22.68
2	17	6.46	2	17	8.76
3	9	3.42	3	9	4.64
4	19	7.22	4	19	9.79
5	69	26.24			
6	105	39.92	6	105	54.12
Total	263	100.00	Total	194	100.00
**Practice model:**			**Practice model:**		
Org_type	Freq.	Percent	Org_type	Freq.	Percent
academic	168	63.88	academic	99	51.03
community private practice	5	1.90	community private practice	5	2.58
medical group employed	90	34.22	medical group employed	90	46.39
Total	263	100.00	Total	194	100.00
**Specialty:**			**Specialty:**		
specialty_pre_8	Freq.	Percent	specialty_pre_8	Freq.	Percent
FAM/MP	55	20.91	FAM/MP	48	24.74
ADULT-GEN	38	14.45	ADULT-GEN	24	12.37
ADULT-SPECIALTIES	46	17.49	ADULT-SPECIALTIES	28	14.43
PEDIATRICS	38	14.45	PEDIATRICS	30	15.46
NEURO/PSYCH	14	5.32	NEURO/PSYCH	10	5.15
OB/GYN	27	10.27	OB/GYN	21	10.82
SURGERY	45	17.11	SURGERY	33	17.01
Total	263	100.00	Total	194	100.00
**Degree:**			**Degree:**		

© 2025 Olson KD et al. *JAMA Network Open*.

<table>
<tr><td>level_pre | Freq. Percent</td><td>level_pre | Freq. Percent</td></tr>
<tr><td>-------------+-----------------------------------</td><td>-------------+-----------------------------------</td></tr>
<tr><td>MD/DO/MBBS | 232 88.21</td><td>MD/DO/MBBS | 179 92.27</td></tr>
<tr><td>NP/PA/APC | 29 11.03</td><td>NP/PA/APC | 14 7.22</td></tr>
<tr><td>other | 2 0.76</td><td>other | 1 0.52</td></tr>
<tr><td>-------------+-----------------------------------
-</td><td>-------------+-----------------------------------</td></tr>
<tr><td>Total | 263 100.00</td><td>Total | 194 100.00</td></tr>
<tr><td></td><td></td></tr>
<tr><td>Practice_years (means):</td><td>Practice_years (means):</td></tr>
<tr><td></td><td></td></tr>
<tr><td>Variable | N Mean</td><td>Variable | N Mean SD</td></tr>
<tr><td>-------------+--------------------</td><td>-------------+----------------------------</td></tr>
<tr><td>practice_y~e | 261 15.09195</td><td>practice_y~e | 192 12.95313 8.185</td></tr>
<tr><td>--</td><td>--</td></tr>
<tr><td></td><td></td></tr>
<tr><td>Years in practice, groups</td><td>Years in practice, groups</td></tr>
<tr><td></td><td></td></tr>
<tr><td>yearsgrps_p |</td><td>yearsgrps_p |</td></tr>
<tr><td>re | Freq. Percent</td><td>re | Freq. Percent</td></tr>
<tr><td>-------------+-----------------------------------</td><td>-------------+-----------------------------------</td></tr>
<tr><td>>=1 to <=5 | 44 16.86</td><td>>=1 to <=5 | 40 20.83</td></tr>
<tr><td>>5 to <=10 | 46 17.62</td><td>>5 to <=10 | 41 21.35</td></tr>
<tr><td>>10 to <=15 | 69 26.44</td><td>>10 to <=15 | 56 29.17</td></tr>
<tr><td>>15 to <=20 | 36 13.79</td><td>>15 to <=20 | 24 12.50</td></tr>
<tr><td>>20 | 66 25.29</td><td>>20 | 31 16.15</td></tr>
<tr><td>-------------+-----------------------------------</td><td>-------------+-----------------------------------</td></tr>
<tr><td>Total | 261 100.00</td><td>Total | 192 100.00</td></tr>
<tr><td></td><td></td></tr>
<tr><td>Sex:</td><td>Sex:</td></tr>
<tr><td></td><td></td></tr>
<tr><td>Gender | Freq. Percent</td><td>Gender | Freq. Percent</td></tr>
<tr><td>-------------+-----------------------------------</td><td>-------------+-----------------------------------</td></tr>
<tr><td>female | 141 53.61</td><td>female | 108 55.67</td></tr>
<tr><td>male | 120 45.63</td><td>male | 86 44.33</td></tr>
<tr><td>notknown | 2 0.76</td><td></td></tr>
<tr><td>-------------+-----------------------------------</td><td>-------------+-----------------------------------</td></tr>
<tr><td>Total | 263 100.00</td><td>Total | 194 100.00</td></tr>
<tr><td></td><td></td></tr>
<tr><td>.</td><td></td></tr>
<tr><td>Documentation style:</td><td></td></tr>
<tr><td></td><td></td></tr>
<tr><td>| Freq. Percent</td><td>Manual typing | Freq. Percent</td></tr>
<tr><td>---------------+-----------------------------------</td><td>---------------+-----------------------------------</td></tr>
</table>

© 2025 Olson KD et al. *JAMA Network Open*.

Manual typing | 218 82.89	Manual typing | 162 83.51
Templates / dot-phrases | 224 85.17	Templates / dot-phrases | 169 87.11
Dictation | 123 46.77	Dictation | 89 45.88
Another ambient solution | 5 1.90	Another ambient solution | 5 2.58
Scribes | 43 16.35	Scribes | 27 13.92
Other | 4 1.52	Other | 4 2.06
------------+-----------------------	------------+-----------------------
Total | 263 100.00	Total | 194 100.00
**Generated >=5 notes with the ambient-ai scribe**	
	five_notes | Freq. Percent
	------------+-----------------------
	0 | 8 4.12
	1 | 186 95.88
	------------+-----------------------
	Total | 194 100.00
.	

© 2025 Olson KD et al. *JAMA Network Open*.

eTable 3. Ten-Point Burnout for Demographic Group, Paired *t* Test Pre and Post Intervention (Means and Differences)

	n	Baseline Mean (SE)	Follow-up Mean (SE)	Difference Mean (SE)	p-value diff>0	p-value diff !=0
**BURNOUT-ONLY SAMPLE**						
Total	194					
**Health System Site:**						
1	44	4.17 (0.30)	3.91 (0.29)	0.26 (0.60)	0.128	0.256
2	17	5.90 (0.55)	4.18 (0.55)	1.72 (0.60)	**0.005**	**0.011**
3	9	4.00 (0.75)	3.50 (0.88)	0.50 (0.63)	0.223	0.447
4	19	5.03 (0.50)	4.32 (0.47)	0.71 (0.42)	0.055	0.111
5	-	-	-	-	-	-
6	97	4.53 (0.53)	4.22 (0.42)	0.30 (0.30)	**0.014**	**0.027**
Missing	0					
**Practice model:**						
academic	91	4.56 (0.20)	4.23 (0.21)	0.32 (0.14)	**0.011**	**0.023**
community private practice	5	4.15 (0.55)	4.15 (0.55)	0 (0.71)	0.500	1.000
medical group employed	90	4.65 (0.23)	4.00 (0.22)	0.65 (0.20)	**<0.001**	**0.001**
Missing	8					
**Specialty:**						
FAM/MP	48	4.75 (0.30)	3.77 (0.29)	0.98 (0.28)	**<0.001**	**<0.001**
ADULT-GEN	23	3.64 (0.42)	3.25 (0.32)	0.39 (0.34)	0.128	0.257
ADULT-SPECIALTIES	27	4.83 (0.33)	4.33 (0.28)	0.50 (0.28)	**0.042**	0.083
PEDIATRICS	27	5.42 (0.42)	5.08 (0.40)	0.33 (0.23)	0.081	0.161
NEURO/PSYCH	9	3.75 (0.50)	3.75 (0.50)	0 (0.38)	0.081	1.000
OB/GYN	19	4.55 (0.40)	3.96 (0.42)	0.59 (0.34)	**0.048**	0.062
SURGERY	33	4.41 (0.37)	4.48 (0.45)	0.07 (0.29)	0.594	0.812
Missing	8					
**Degree:**						
MD/DO/MBBS	179	4.64 (0.15)	4.12 (0.15)	0.52 (0.12)	**<0.001**	**<0.001**
NP/PA/APC	14	3.94 (0.59)	4.12 (0.65)	0.17 (0.31)	0.701	0.584
other	1					
Missing	0					
**Years in practice, groups**						
>=1 to <=5	38	4.67 (0.35)	4.43 (0.34)	0.24 (0.20)	0.127	0.254
>5 to <=10	40	4.49 (0.29)	4.26 (0.34)	0.23 (0.24)	0.176	0.352
>10 to <=15	54	4.38 (0.30)	4.00 (0.28)	0.38 (0.22)	**0.048**	0.095
>15 to <=20	24	4.94 (0.34)	3.81 (0.36)	1.13 (0.41)	**0.006**	**0.011**
>20	38	4.86 (0.40)	3.97 (0.37)	0.88 (0.32)	**0.005**	**0.009**
Missing	0					

© 2025 Olson KD et al. *JAMA Network Open*.

**Sex:**						
female	102	5.08 (0.20)	4.62 (0.20)	0.46 (0.17)	**0.004**	**0.009**
male	84	4.00 (0.20)	3.52 (0.20)	0.48 (0.16)	**0.002**	**0.003**
Missing	8					
Unadjusted pre- and post-intervention scores reported as means (standard error, SE) and arithmetic difference of means (SE) transformed to 10-point scales.						

© 2025 Olson KD et al. *JAMA Network Open*.
