# Supplementary material for: Use of Ambient AI Scribes to Reduce Administrative Burden and Professional Burnout
Source: JAMA Netw Open. 2025 Oct 2;8(10):e2534976. doi: 10.1001/jamanetworkopen.2025.34976 (PMC12492056; doi:10.1001/jamanetworkopen.2025.34976)
Supplement: Supplement 2. — Data Sharing Statement [file jamanetwopen-e2534976-s002.pdf]

Data Sharing Statement

Data
Data available: No

Additional Information
Explanation for why data not available: Upon reasonable request made to the corresponding author, the data are available for further research from any of the participating health systems that agree to share their contributions to the data set.
